# Tracing Photoinduced Hydrogen Migration in Alcohol Dications from Time-Resolved Molecular-Frame Photoelectron Angular Distributions

**DOI:** 10.1021/acs.jpca.3c07640

**Published:** 2024-02-07

**Authors:** T. Kuraoka, S. Goto, M. Kanno, S. Díaz-Tendero, J. Reino-González, F. Trinter, A. Pier, L. Sommerlad, N. Melzer, O. D. McGinnis, J. Kruse, T. Wenzel, T. Jahnke, H. Xue, N. Kishimoto, K. Yoshikawa, Y. Tamura, F. Ota, K. Hatada, K. Ueda, F. Martín

**Affiliations:** † Department of Physics, University of Toyama, Gofuku 3190, Toyama 930-8555, Japan; ‡ Department of Chemistry, Tohoku University, 6-3 Aramaki Aza-Aoba, Aoba-ku, Sendai 980-8578, Japan; § Departamento de Química, Universidad Autónoma de Madrid, Madrid 28049, Spain; ∥ Condensed Matter Physics Center (IFIMAC), Universidad Autónoma de Madrid, Madrid 28049, Spain; ⊥ Institute for Advanced Research in Chemical Sciences (IAdChem), Universidad Autónoma de Madrid, Madrid 28049, Spain; # Instituto Madrileño de Estudios Avanzados en Nanociencia (IMDEA-Nano), Campus de Cantoblanco, Madrid 28049, Spain; ¶ Molecular Physics, Fritz-Haber-Institut der Max-Planck-Gesellschaft, Faradayweg 4-6, Berlin 14195, Germany; ∇ Institut für Kernphysik, Goethe-Universität Frankfurt, Max-von-Laue-Straβe 1, Frankfurt am Main 60438, Germany; ○ Max-Planck-Institut für Kernphysik, Saupfercheckweg 1, Heidelberg 69117, Germany; ⧫ European XFEL, Holzkoppel 4, Schenefeld 22869, Germany

## Abstract

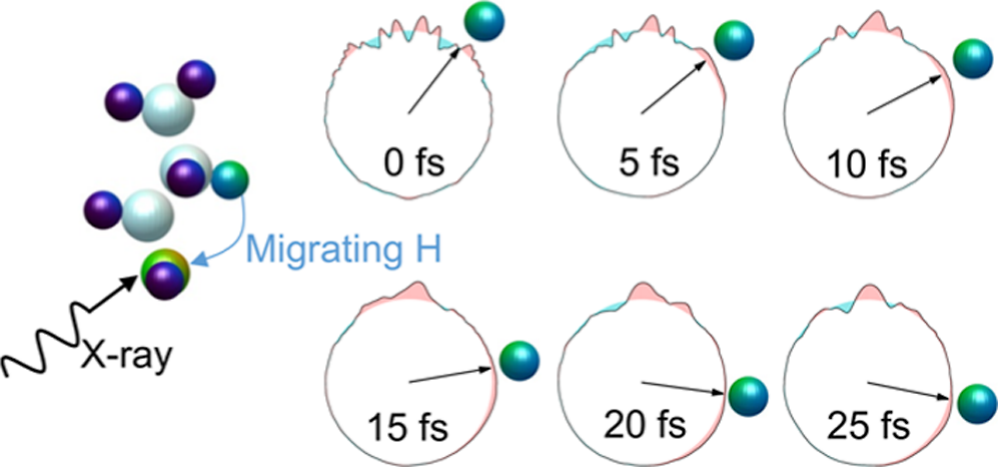

The recent implementation of attosecond and few-femtosecond X-ray pump/X-ray probe schemes in large-scale free-electron laser facilities has opened the way to visualize fast nuclear dynamics in molecules with unprecedented temporal and spatial resolution. Here, we present the results of theoretical calculations showing how polarization-averaged molecular-frame photoelectron angular distributions (PA-MFPADs) can be used to visualize the dynamics of hydrogen migration in methanol, ethanol, propanol, and isopropyl alcohol dications generated by X-ray irradiation of the corresponding neutral species. We show that changes in the PA-MFPADs with the pump–probe delay as a result of intramolecular photoelectron diffraction carry information on the dynamics of hydrogen migration in real space. Although visualization of this dynamics is more straightforward in the smaller systems, methanol and ethanol, one can still recognize the signature of that motion in propanol and isopropyl alcohol and assign a tentative path to it. A possible pathway for a corresponding experiment requires an angularly resolved detection of photoelectrons in coincidence with molecular fragment ions used to define a molecular frame of reference. Such studies have become, in principle, possible since the first XFELs with sufficiently high repetition rates have emerged. To further support our findings, we provide experimental evidence of H migration in ethanol−OD from ion–ion coincidence measurements performed with synchrotron radiation.

## Introduction

Attosecond chemistry is a rapidly evolving field aiming at visualizing electronic motion in molecules and eventually controlling that motion by acting on the electrons before the nuclei have time to respond.^1,2^ In this way, one hopes to modify the chemical behavior of molecules, going beyond the usual approaches in femtochemistry. Controlling electronic motion requires an attosecond time resolution. However, light nuclei, such as protons, can move significantly in just a few femtoseconds. Therefore, imaging this motion may also require high temporal resolution, even attosecond resolution. This is the case for hydrogen migration, one of the most fundamental processes in chemistry and biology.^3−7^ Attosecond pulses are thus the ideal tool to investigate such motion.

Attosecond pulses are nowadays produced in the laboratory from high-harmonic generation from noble gases^8−10^ and in large-scale X-ray free-electron laser (XFEL) facilities.^9,11^ Concerning the imaging of rapid structural changes, current efforts focus on improving existing and developing new visualization tools^12−14^ that can unambiguously provide such information in spite of the many nuclear degrees of freedom inherent to most molecules. Coulomb explosion imaging (CEI)^15−27^ and laser-induced electron diffraction (LIED)^28−31^ have been successfully used to investigate hydrogen motion in a variety of molecules, including hydrogen migration and roaming, but both have inherent limitations. CEI is not yet a mature approach, which requires detecting several ionic fragments in coincidence; otherwise, the sought-for dynamics may remain hidden. Ideally, a CEI measurement consists of a full fragmentation of the molecule into atomic species and a corresponding detection of all of these ions. However, recent studies indicate that the latter requirement can be relaxed and a rather complete image can be obtained already from subsets of the generated ions.^26^ LIED can only retrieve the dynamics occurring during the optical cycle of the driving laser, so imaging is limited to the very early stages of the migration process. In a previous theoretical work,^32^ an alternative approach to image rapid structural changes of molecules has been proposed: time- and momentum-resolved photoelectron diffraction (TMR-PED). This method requires the use of two ultrashort X-ray pulses. The first one (pump) is used to ionize the K-shell of the constituting atoms and to produce dications via Auger-Meitner decay, and the second one (probe) is used to generate K-shell photoelectrons for the PED measurement as a function of the pump–probe delay. As molecules in the gas phase are randomly oriented in space, it is necessary to measure their spatial orientation in order to determine the full three-dimensional photoelectron diffraction pattern in the molecular frame of reference. This can be done by measuring, in coincidence, the momenta of ionic fragments (which were generated by the probe pulse). Experimental approaches such as, e.g., a COLTRIMS reaction microscope^33,34^ are capable of achieving that task and measuring, in addition, the photoelectron momentum in coincidence. The work of Ota et al.^32^ showed that TMR-PED obtained in a (molecular) reference frame that is appropriate for multicoincidence detection allows for direct imaging of single- and double-hydrogen migration in doubly charged ethanol with both few-fs and Å resolutions. The signature of the migrating hydrogen atoms was observed in the polarization-averaged molecular-frame photoelectron angular distributions (PA-MFPADs) in the form of a moving feature that reflects the average trajectory followed by the H atoms, thus allowing for a straightforward visualization of the dynamics in real space. In this respect, it is worth noticing that PA-MFPADs usually provide a more direct image of the molecular geometry than MFPADs.^32,35−38^

In the present work, we further analyze the validity of TMR-PED to image H migration in three other alcohols, methanol, 1-propanol (propanol from now on), and 2-propanol (isopropanol from now on), and compare the calculated PA-MFPADs with those previously reported for ethanol. We show that, in all cases, H migration leads to a moving trace in the PA-MFPADs, especially during the first 10–20 fs. We also show that from these observables one can retrieve the H migration times for each system. We discuss the differences in the dynamics of H migration observed in the three systems and what one could expect in larger alcohols. Finally, to give some additional hints about the experimental feasibility of the proposed method, we present the results of synchrotron-radiation measurements on ethanol−OD molecules, in which fragment ions have been measured in coincidence. The resulting two-dimensional ion–ion coincidence maps unambiguously show the presence of a H migration. However, time-resolved studies as presented here are not possible at synchrotrons due to their inherent long pulse duration. As indicated before, the first X-ray free-electron laser facilities started to provide the shortest X-ray pulses at repetition rates, which finally allowed for performing (multi)coincidence measurements.^39−41^ Therefore, our theoretical predictions will be useful to guide future experiments in these facilities and provide new insight into the dynamics of the H migration process.

## Principle of Measurement

**Figure 1:**
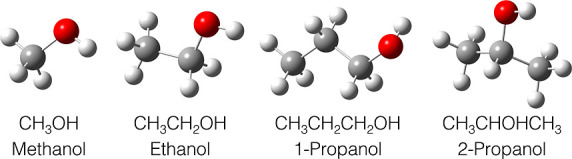
Molecular structures.

**Figure 2:**
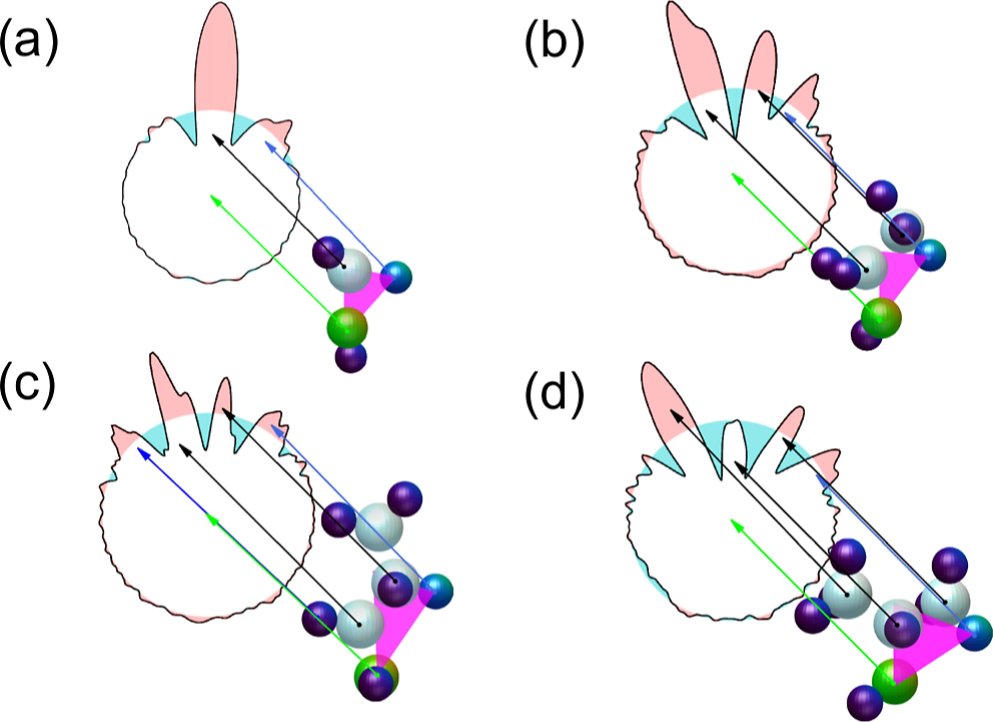
Definition of the reaction plane in the four molecules investigated in this work. For single-hydrogen migration, this plane contains the triangle (in pink) whose vertices coincide with the centers of mass of , OH^+^, and the migrating H atom. Color code for the different atoms: oxygen—green, carbon—white, migrating hydrogen atom—pale blue, and other hydrogen atoms—dark blue. The panel also shows the projection of the PA-MFPAD on the chosen plane. (a) Methanol. (b) Ethanol. (c) Propanol. (d) Isopropanol.

The three-dimensional structures of the four molecules considered in this work are shown in Figure 1. All of them are composed of C and H atoms and include a single hydroxyl group, –OH, which gives them the alcoholic character. Our example of TMR-PED to reveal the dynamics of hydrogen migration in the doubly charged molecular dications consists of using a pump–probe scheme with two ultrashort X-ray pulses. The pump pulse will ionize either the C 1s or the O 1s shells of the neutral molecules, leading to molecular monocations, which will subsequently decay to the corresponding molecular dications by emitting an Auger–Meitner electron. The probe pulse will be used to produce an O 1s photoelectron, which will then be diffracted by the surrounding H and C atoms. Variation of the pump–probe delay will thus provide temporal variation of the PED pattern. As discussed in ref (32), the reason to look at O 1s ionization by the probe pulse is that the migrating hydrogen atom H_mig_ will preferentially be caught by the oxygen atom. After ionization by the probe pulse, the resulting molecular trication will further ionize as a result of Auger−Meitner decay, thus leading to a molecular tetracation that will Coulomb-explode, leading to singly charged fragments or to a doubly charged and two singly charged fragments or to two doubly charged ones. We will focus on the complex [H_mig_···OH^+^] that is formed after the pump step. In this complex, the distance between H_mig_ and OH^+^ is expected to become progressively shorter until an OH_2_^+^ group is formed. As a result of the ionization of the O 1s orbital and the Auger−Meitner decay following the probe step, this complex will acquire two additional positive charges, leading to H_mig_^+^, O^+^, and H^+^. The full sequence of events while the H atom is still migrating can thus be described as
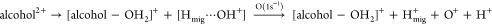
1where  denotes the molecular species that result when the original alcohol^2+^ dication loses an H atom and OH^+^. Therefore, experimentally, the reaction plane must be determined by measuring the momentum correlation between H_mig_^+^, , and the sum of O^+^ and H^+^. The reaction planes for the four investigated molecules are depicted in Figure 2. All polarization-averaged molecular-frame photoelectron angular distributions reported below refer to this choice of the reaction planes.

## Theoretical Methods

### Molecular Dynamics Simulations

Molecular dynamics (MD) calculations have been performed for the methanol, ethanol, propanol, and isopropanol dications using the atom-centered density matrix propagation (ADMP) method.^42−44^ In these simulations, the nuclei move classically in the potential computed on-the-fly with density functional theory (DFT). Initial internal energies have been chosen to be 5 eV for methanol, 10 eV for ethanol, and 15 eV for propanol and isopropanol. These values are comparable in magnitude to those used in previous work and correspond to typical electronic excitation energies of doubly charged molecular ions produced by the interaction with strong IR pulses^23,24,45^ or fast atomic ions,^46−48^ or produced after Auger−Meitner decay of the singly charged molecular ion resulting from X-ray ionization.^32,48,49^ The specific values chosen in the present work allow for a meaningful comparison between the different systems since the available energy per atomic mass unit is comparable for all of them (0.16, 0.22, and 0.25 eV/amu, respectively) and reflects the ability of the larger dications to store more energy while remaining unbroken. In these simulations, we are implicitly assuming that all or part of the electronic excitation energy after Auger–Meitner decay is rapidly transferred to the nuclear degrees of freedom (vibrational, rotational, and kinetic) well before H migration and fragmentation of the molecular dications occur. This would be of course a severe approximation if we were interested in the very early states of the coupled electronic and nuclear motion (i.e., the first few fs), but it should be acceptable at the longer times we are interested in this work, as shown, e.g., in refs (23), (24), (45)–^47^, (50)–^53^, where this approximation has been successfully used to explain a variety of experiments. With this internal energy, which is randomly distributed among the nuclei, the latter start moving in the molecular potential. Typically, we have chosen 1000 initial conditions with random initial momenta. MD and potential-energy-surface calculations have been performed using the Gaussian16 package.^54^

To keep computational effort within reasonable limits, we have considered only singlet spin configurations for all molecular dications. We have used the B3LYP functional.^55−57^ For methanol, we have chosen the 6-311G(2d,d,p) (CBSB7) basis set,^58−60^ and for ethanol, propanol, and isopropanol, we chose the 6-31++G(d,p) basis set.^59−61^ Propagations were performed with a time step of Δ*t* = 0.1 fs and a fictitious electron mass of μ = 0.1 amu, ensuring adiabaticity in the simulations. For ethanol, propanol, and isopropanol, the different structural isomers existing at room temperature were considered,^62,63^ namely the gauche and trans conformers for ethanol,^64−66^ the gauche and trans conformers for 2-propanol,^67,68^ and four conformers combining trans and gauche configurations around the C–C and C–O bonds for 1-propanol.^69,70^

### PA-MFPAD Calculations

To calculate the electronic continuum states that are necessary to obtain PA-MFPADs, we have used the multiple-scattering method of ref (71). In this method, a multicenter expansion in spherical harmonics and a numerical solution of the local Schrödinger equation on each atomic site are performed. Also, the scattering potentials are described within the muffin-tin approximation. Implementation of this methodology in the four molecules considered in this work is similar to the one used in ref (32) for ethanol, so only a brief description will be given here. For all molecules, we have considered two values of the photoelectron energy: 100 eV and 2.5 keV. For 100 eV, the maximum values of the angular momentum, *l*_max_, for the partial-wave expansion were 5, 5, and 4 for O, C, and H, respectively, and for 2.5 keV, 10 for all of them. These continuum wave functions were used to evaluate the ionization probabilities for the three Cartesian components of the electric dipole by using first-order perturbation theory and the dipole approximation. All calculations were performed by using the MsSpec code.^72^

## Results and Discussion

From the MD simulations, we estimated the relative yields of single H migration for each molecule: approximately 4% for methanol, 4% for ethanol, 8% for propanol, and 5% for isopropanol. This partially reflects the fact that as the size of the molecular dication increases, more H atoms are available to migrate to the OH group. In the case of isopropanol, as the OH group is in the central part of the molecule, fragmentation leading to CH_3_^+^ is more likely than for the other three alcohols, so that the amount of H migration is smaller than for propanol. The calculated yields were obtained at 100 fs, which is long enough for H migration to be completed. These yields include all possible channels in which a hydrogen atom has migrated and therefore include dissociative and nondissociative hydrogen-migration channels. To obtain the ratio between the latter two, one should consider longer propagation times since fragmentation channels involving the breakup of covalent bonds between the heavier atomic species may be much slower. However, this is irrelevant for the purposes of the present work.

**Figure 3:**
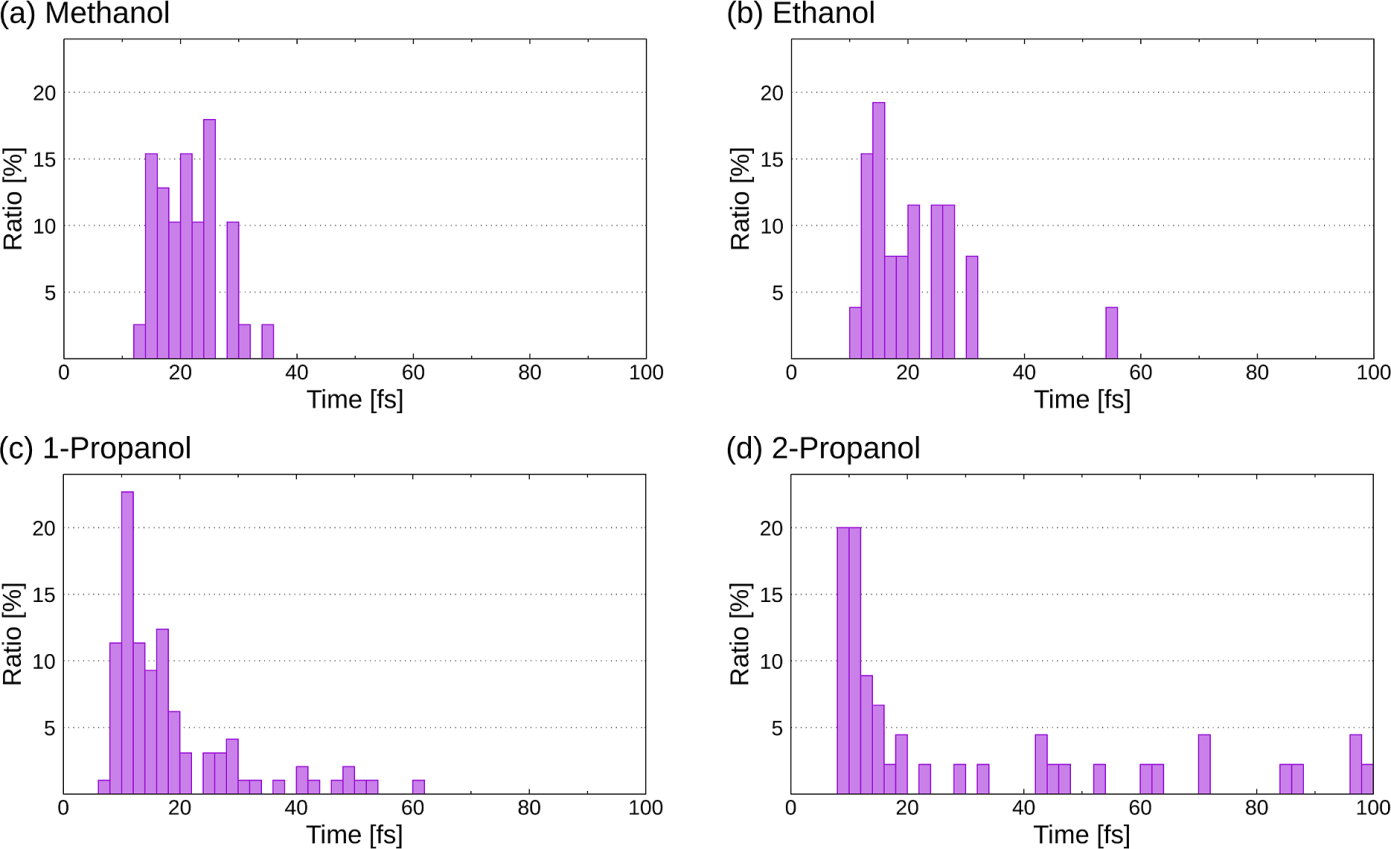
Time histograms showing the number of trajectories leading to H migration in intervals of 2 fs. The results have been normalized to the total number of trajectories associated with single H migration for each particular system. (a) Methanol. (b) Ethanol. (c) Propanol. (d) Isopropanol.

For each trajectory, we identified the time at which H migration is completed. Figure 3 depicts time histograms showing the number of trajectories leading to H migration at intervals of 2 fs. For a better comparison between the different species, for each system, the number of trajectories at each time interval has been normalized to the total number of trajectories associated with a single H migration for that particular system. As can be seen, for methanol and ethanol, the temporal distributions are qualitatively similar, with a maximum at around 20 fs and a width of approximately ±10 fs. For propanol and isopropanol, however, they consist of a peak at around 10 fs, which is narrower than for methanol and ethanol, plus a tail that extends up to 3 to 5 times longer. That the distributions for propanol and isopropanol are broader is not surprising, since these molecules have H atoms farther from OH than in methanol and ethanol and, therefore, need more time to travel. The difference in the position of the maximum (10 fs vs 20 fs) is certainly due to the fact that the initial internal energy used to perform the calculations is higher for the larger molecular dications than for the smaller ones. Although the internal energy available per atom is comparable in all four molecules, in reality, the total energy is not equally redistributed among all atoms, as the lighter ones (hydrogen), being more mobile, can acquire a larger fraction of this energy.

**Figure 4:**
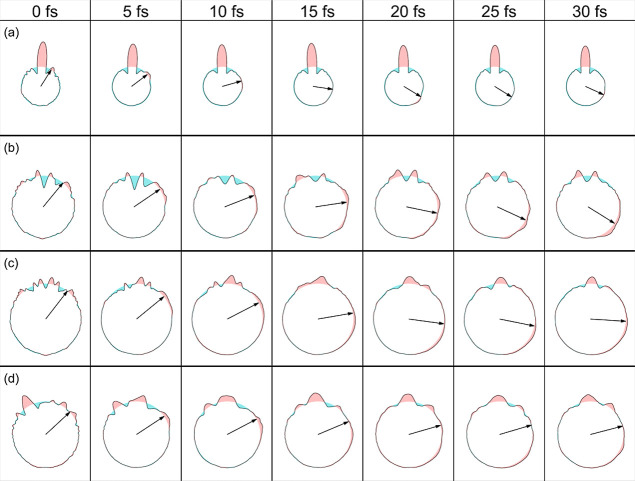
Snapshots of PA-MFPADs averaged over all trajectories leading to H migration for a photoelectron energy of 2.5 keV. The arrows point to the moving feature associated with H migration. (a) Methanol. (b) Ethanol. (c) Propanol. (d) Isopropanol. The arrows indicate the position of the migrating H atom after averaging over all trajectories leading to hydrogen migrations.

**Figure 5:**
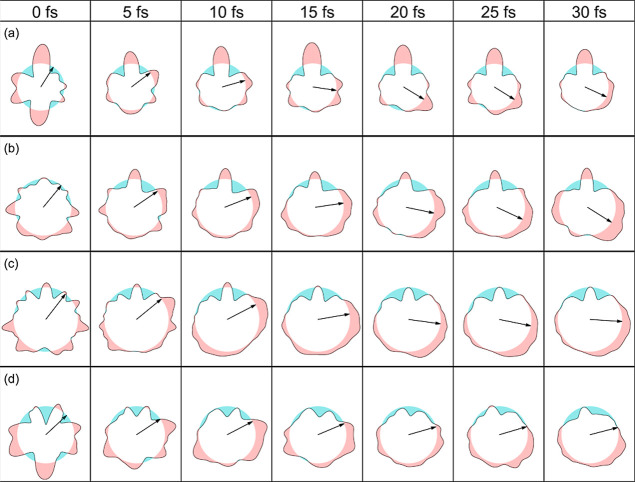
Snapshots of PA-MFPADs averaged over all trajectories, leading to H migration for a photoelectron energy of 100 eV. (a) Methanol. (b) Ethanol. (c) Propanol. (d) Isopropanol. The arrows indicate the position of the migrating H atom after averaging over all trajectories leading to hydrogen migrations.

Figures 4 and 5 show the temporal evolution of the PA-MFPADs in the chosen reaction plane for a photoelectron energy of 2.5 keV and 100 eV, respectively. The complete movies are given in the Supporting Information. The results have been obtained by averaging over the PA-MFPADs associated with each single trajectory. The arrows indicate the position of the migrating H atom after averaging over all trajectories leading to hydrogen migrations. As can be seen, diffraction of the O 1s photoelectron by the neighboring atomic centers leads to lobes or depletions with respect to a perfectly isotropic distribution (which is shown as a circle). As expected, these features are more pronounced at 100 eV than at 2.5 keV since slower electrons are more sensitive to the details of the multicentric molecular potential. The features change as the different atoms move with respect to the reaction plane, but those associated with H migration roughly follow the motion of the hydrogen atom, especially at a photoelectron energy of 2.5 keV. We note, however, that at longer times (>15–20 fs), the position of the arrows more or less stabilizes while the corresponding lobe keeps moving. This is due to the fact that, at longer times, H migration has finished for some trajectories but not for others, so that the position of the migrating H atom obtained by averaging over all trajectories (represented by the arrow) does not exclusively reflect the dynamics of the H atoms that are still migrating. Remarkably, although the averaging over different trajectories is responsible for some blurring of the lobes and depletions that would be observed from a single trajectory, hydrogen migration can still be associated with one of the moving features. There are, however, significant differences between the different systems. In methanol, the moving lobe is very well localized, especially at 2.5 keV, which makes the identification of the H migration easier. The price to pay is that the size of this lobe is pretty small. For the other three systems, delocalization of the moving lobe increases with size, making it more difficult to assign an average trajectory to the H migration process. This is the result of the increasing diversity of migration pathways with molecular size. Therefore, the association of H migration to a specific moving feature in the PA-MFPAD and, hence, to an average trajectory becomes more ambiguous for the larger molecules. This is less dramatic at the highest energy, 2.5 keV, although, as for methanol, the moving features are much less pronounced. From these results, one can expect that the signature of H migration in the PA-MFPADs will become even less clear in larger alcohols.

### Experimental Findings

Currently available technology at XFELs allows, in principle, to perform experiments as the ones we propose in this work,^39^ and corresponding studies are expected for the near future. In order to give additional support for the feasibility of measuring the hydrogen-migration processes, we performed a synchrotron experiment on ethanol–OD molecules. The use of a deuterated sample simplifies the atomic assignment in the experiment without modifying the basic concepts described above, though at the price of slowing down the migration dynamics due to the larger mass of deuterium. The latter has no practical consequence for performing the experiment, although theoretical simulations would be more expensive since larger integration times should be used. In these measurements, the molecular dication was generated by ionizing the oxygen K-shell via absorption of photons with an energy of 553 eV, which is followed by Auger–Meitner decay yielding the desired C_2_H_5_OD^2+^ species. These may fragment or be subject to hydrogen migration. We identify the latter cases in a first step by measuring molecular fragment ions in coincidence and examining the corresponding photoion–photoion coincidence (PIPICO) map. In a second step, in order to cleanly identify the final state of a hydrogen-migration event and to discriminate these comparably weak channels from the background, we performed a full three-dimensional momentum analysis of the recorded ions. The sum momentum of the measured ions plays a particular role in this analysis, as it can be inspected in order to differentiate between different molecular breakup channels and true coincidences from false coincidences (i.e., from background).^34^ If all molecular fragments generated in the photoreaction are detected by coincidence, the sum momentum consists of the initial momentum of the molecule prior to the reaction, the linear momentum imparted by the photon, and the recoil momentum of the emitted electrons. The last two contributions are small and can be neglected for the purpose of this analysis. If the sum momentum is examined in the center-of-mass frame of the molecule, then the first contribution is by definition zero as well. Therefore, restricting the measured data set to cases where the sum momentum of the measured fragments (in the center-of-mass frame) is close to zero will provide a clean signal of the targeted breakup channel. In the present case, we employed a corresponding gate which requires |*p*_sum_| < 20 a.u. for a photoionization event to be flagged as valid. In case the molecule breaks into, e.g., three fragments (and only two of them are detected), the sum momentum of these two corresponds to the recoil of the third particle onto the center of mass. If the third fragment consists only of a proton, its recoil on the other two (much heavier) fragments is typically small. Accordingly, if only the two heavier fragments are measured in coincidence, a gate requiring |*p*_sum_| < 20 a.u. is still a reasonable choice to cleanly extract such a fragmentation channel from the data set.

**Figure 6:**
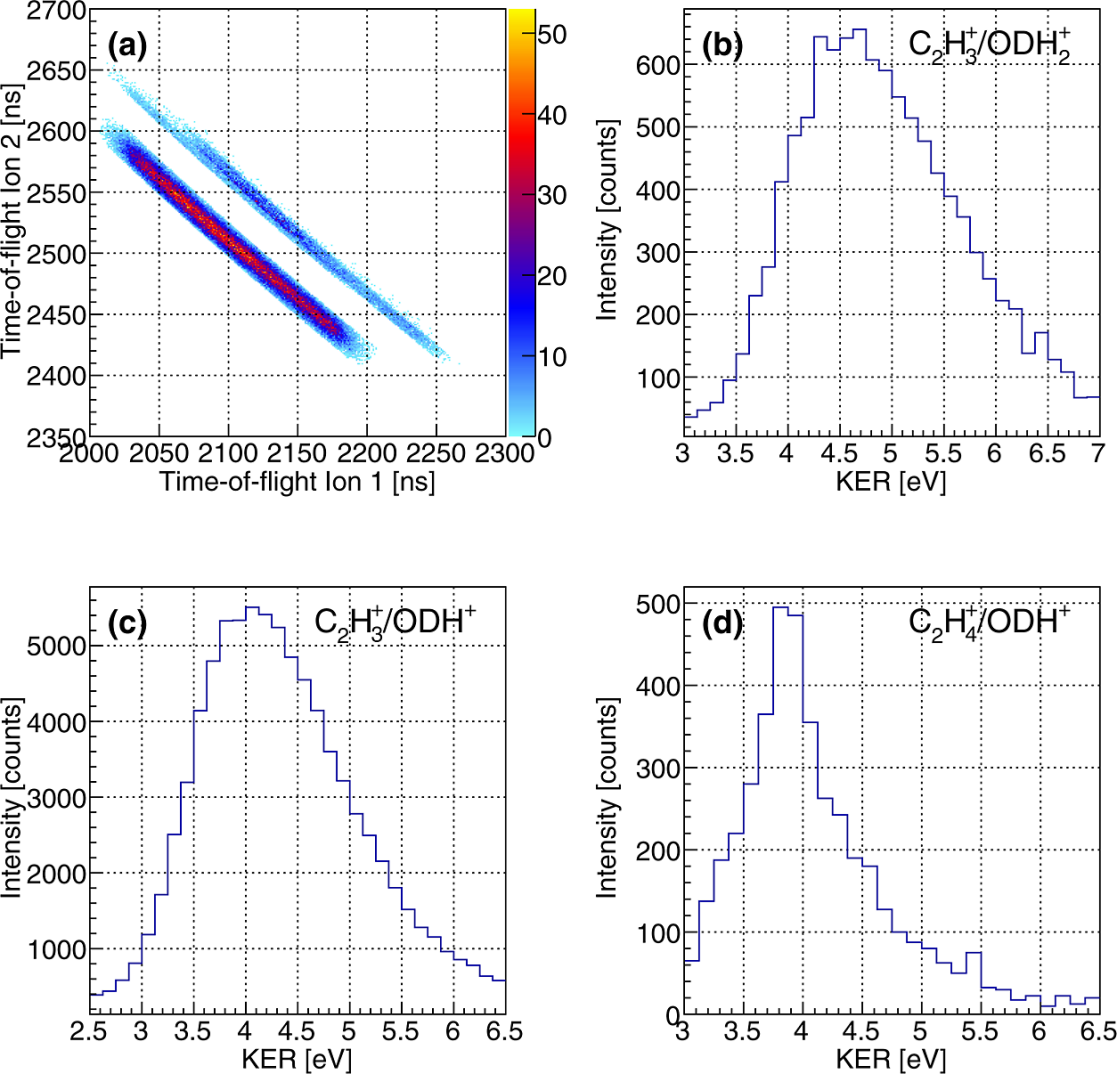
(a) Photoion–photoion coincidence map (after filtering on the sum momentum of the measured ions, see text) showing the features belonging to a breakup of the ethanol–OD molecule into C_2_H_3_^+^/ODH_2_^+^, C_2_H_3_^+^/ODH^+^, and C_2_H_4_^+^/ODH^+^. (b–d) Measured kinetic energy release for the corresponding breakup channels, i.e., for (b) C_2_H_3_^+^/ODH_2_^+^, (c) C_2_H_3_^+^/ODH^+^, and (d) C_2_H_4_^+^/ODH^+^.

Figure 6a shows a PIPICO histogram, where the flight time of a measured first ion is plotted versus that of a detected second ion. Different breakup channels appear as line-shaped islands in this type of histogram. Figure 6a shows, at first glance, two distinct features. The stronger of the two corresponds to a breakup of the molecule into a C_2_H_3_^+^/ODH^+^ ion pair. The less pronounced island consists of two partially overlapping fragmentation channels, i.e., C_2_H_4_^+^/ODH^+^ and C_2_H_3_^+^/ODH_2_^+^. Note that the aforementioned gating on |*p*_sum_| was performed to enhance the visibility of the fragmentation channels. While the two latter fragmentation channels partially overlap in the PIPICO representation, they are still separable in the full three-dimensional momentum analysis, which yields the kinetic energy release of the three channels, as shown in Figure 6b–d. The synchrotron data cleanly demonstrates that fragmentation channels occur, which can only be generated by single- or double-hydrogen migration in the dicationic state of the ethanol–OD molecule. We summarize the abundance of some of these channels in Table 1, which consists of a breakup of the molecule into ODH^+^ or ODH_2_^+^ fragments. We did not observe cases where the deuterium detached from the oxygen atom and reattached to the other part of the molecule.

**Table 1:** Relative Abundance of Different Breakup Channels of the Ethanol–OD Molecule After Irradiation With Photons of *h*ν = 553 eV^a^

breakup channel	relative abundance
CH_3_^+^/COH_2_D^+^	1.00
C_2_H_4_^+^/ODH^+^	0.08
C_2_H_3_^+^/ODH^+^	1.32
C_2_H_3_^+^/ODH_2_^+^	0.17

a The abundance is given relative to that of a fragmentation of the molecule into CH_3_^+^/COH_2_D^+^.

## Conclusions

We have performed molecular-dynamics and electron-scattering calculations to show how polarization-averaged molecular-frame photoelectron angular distributions (PA-MFPADs) can be used to visualize the dynamics of hydrogen migration in methanol, ethanol, propanol, and isopropanol derivatives generated by X-ray irradiation of the corresponding neutral species. In principle, such PA-MFPADs can be experimentally obtained employing state-of-the-art X-ray free-electron lasers by using an X-ray pump/X-ray probe scheme with few-fs temporal resolution in combination with multicoincidence detection of the photoelectron and the molecular fragments generated by the probe pulse. We show that changes in the positions of some of the peaks arising in the PA-MFPADs as a result of intramolecular photoelectron diffraction reflect the dynamics of hydrogen migration in real space. This is so in spite of the diversity of the trajectories that the migrating H atom can follow on its way to the OH group. We show that visualization of this dynamics is clearer in the smaller systems, methanol and ethanol, due to the smaller number of degrees of freedom. Still, one can also recognize the signature of that motion in propanol and isopropyl alcohol and assign a tentative path to it. To further support the experimental feasibility of the proposed methodology, we also performed synchrotron-radiation experiments in which ionic fragments following ionization and Auger–Meitner decay of ethanol−OD were detected in coincidence. These experiments unambiguously show the existence of ODH^+^ in a meaningful proportion, which can only be explained if an H atom directly bonded to a C atom migrates to the OD group. We hope that the present work will spur current experimental efforts at the newly developed beamlines at XFELs.

## Supporting Information Available

The following files are available free of charge: The Supporting Information is available free of charge at https://pubs.acs.org/doi/10.1021/acs.jpca.3c07640. This material is available free of charge via the internet at http://pubs.acs.org/.

Evolution of the PA-MFPADs in Methanol (MP4)
Evolution of the PA-MFPADs in Ethanol (MP4)
Evolution of the PA-MFPADs in Propanol (MP4)
Evolution of the PA-MFPADs in Isopropanol (MP4)
